# Visual diagnosis: Enucleation status post gunshot wound to the head: A visual diagnosis/case report

**DOI:** 10.1186/1865-1380-4-61

**Published:** 2011-10-03

**Authors:** Bobby Desai, Benjamin Mahon

**Affiliations:** 1 University of Florida Department of Emergency Medicine P.O. Box 100186 Gainesville, 32610, FL, USA

## Abstract

We present the case of a patient who attempted to commit suicide via a gunshot to the head. However, instead of ending his life, he destroyed both of his eyes. Computed tomography scans are shown.

## Background

Patients that attempt suicide are common in the Emergency Department. Suicidal gestures such as intentional medication or illicit drug overdose and attempted laceration of arteries are frequently seen. True intent to commit suicide includes gunshot wounds to the head. These typically are non-survivable injuries, but there occasionally are those that survive these injuries, and we present such a case.

A 55-year-old male presented to our Emergency Department after reportedly shooting himself through the left temple with a.22 caliber handgun in a purported attempt to commit suicide. Per report, the patient was found in his house by a friend, but was easily arousable with intact mentation approximately 20 h after the event allegedly occurred. Upon arrival he reported only moderate facial pain and complete absence of vision, including light and shadow. He denied dizziness, lightheadedness, or confusion.

In the Emergency Department, the patient's vital signs were temperature 37.2 °C, pulse 82 beats per minute, respiratory rate of 20 per minute, and blood pressure 126/60 mmHg. His airway was patent with bilateral breath sounds that were clear, and he had unlabored breathing. He had equal pulses present and strong bilaterally, with regular rate and rhythm on cardiac exam. His abdomen was non-tender and non-distended.

He had extensive bandaging placed by EMS, and after it was removed from around the wound area, his HEENT exam revealed the patient had extensive bilateral periorbital edema with severe ecchymosis, with desiccated tissue remnants of the right globe protruding from the orbital socket. The left globe was complete eviscerated. There was profound edema of the mid-face, but surprising stability of this region on exam. There was a through-and-through wound entering at the left temple, 1 cm in diameter, with a right temple exit wound about 2 cm in diameter, with tissue avulsion. The nasal bridge was intact, without blood in the nares. The tympanic membranes were intact bilaterally without hemotympanum.

On neurological exam, the patient was moving all extremities equally bilaterally with no focal sensory or neurological deficits. Cranial nerves two, three, four, and six could not to be assessed because of complete enucleation of both eyes. Sensation was intact in the bilateral distributions of V1, V2, and V3. He was alert, awake, and oriented to person, place, and time, in no apparent distress, with a Glasgow Coma Scale of 12, with three points off the GCS for visual. The patient's mental status, mood, and affect were appropriate.

Neurosurgery, Oral Maxillofacial Surgery, and Ophthalmology were all emergently consulted.

**Figure 1:**
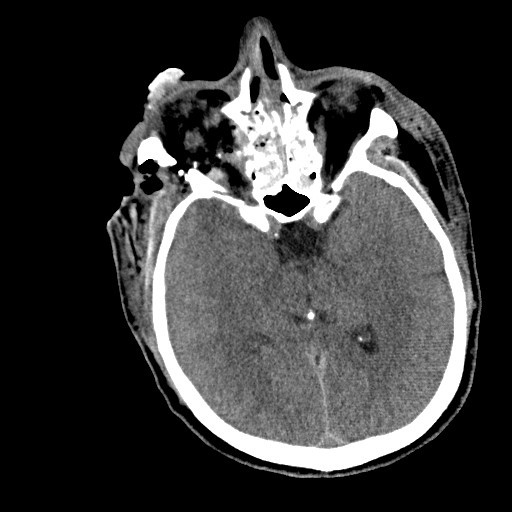
**Right and left orbit extensive damage, CT cuts in sequential order**.

**Figure 2:**
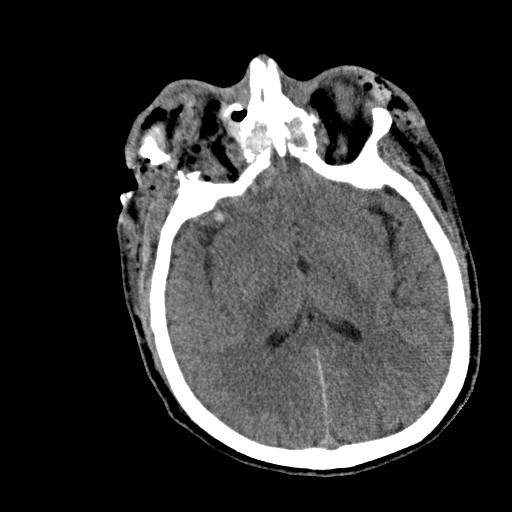
**Right and left orbit extensive damage, CT cuts in sequential order**.

**Figure 3:**
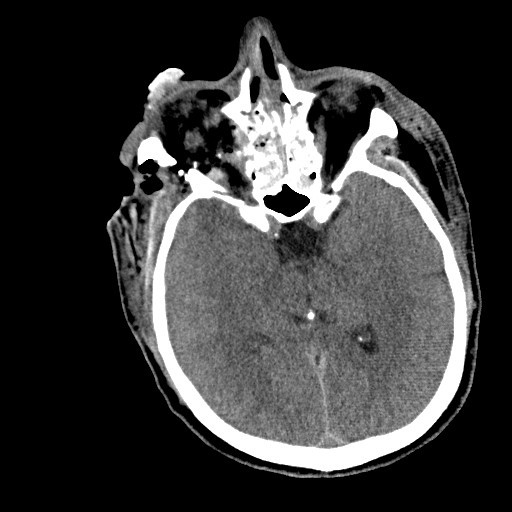
**Right and left orbit extensive damage, CT cuts in sequential order**.

**Figure 4:**
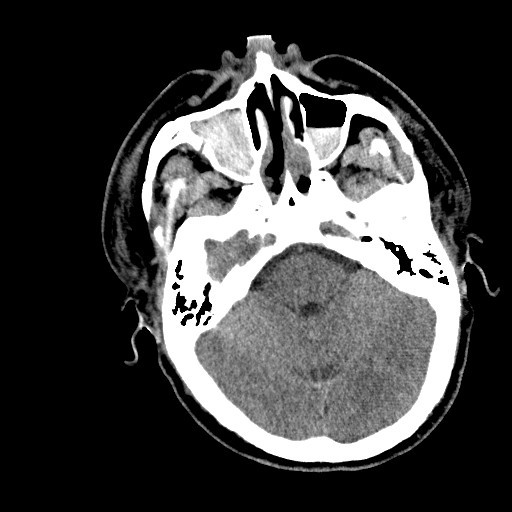
**Extensive hemorrhage into sinus cavities**.

CT of the head, maxillofacial area, and cervical spine with 3D reconstructions were obtained at that time once the patient was deemed clinically stable and suitable for transport (Figures 1, 2, 3 and 4).

### Radiology reported

"Devastating gunshot injury to the maxillofacial region with complete destruction of the globes bilaterally, with multiple bony fragments and air within the retro-orbital regions bilaterally, with fractures through the anterior frontal maxillary region involving both orbits and the maxillary and ethmoid sinuses. There is a comminuted displaced fracture involving the superior orbital wall and frontal sinus on the right with a tiny amount of pneumocephalus. There are comminuted displaced fractures involving nearly every orbital wall."

Neurosurgical evaluation at that time determined that no surgical intervention was needed for the small amount of pneumocephalus. They recommended prophylactic Phenytoin for seizures, and close observation for possible future meningitis.

Ophthalmology reported that there was no chance of recovery of vision, and simply recommended wound care and bacitracin.

The patient was then admitted to the Oral Maxillofacial Service, later receiving open reduction with internal fixations of the right superior orbital rims, the right zygomatic complex, the right zygomatic arch, and reconstruction of the orbital floor, along with obliteration of the frontal sinus with abdominal fat graft placement.

On postoperative day 3 the patient was deemed stable, started on Celexa, and transferred to Psychiatry's local inpatient rehabilitation facility.

## Discussion

In the ED it is not uncommon to see many different variations of suicide attempts, including self-inflicted gunshot wounds, the laceration of arteries, intentional drug overdose, and even self-neglect. However, this case highlights a common, but infrequently discussed, phenomenon, namely, the "botched" suicide.

In many cases, as the one above illustrates, the lay person has an incomplete understanding of anatomy, and fails to appreciate the precise angle and trajectory required to successfully complete a suicide with a gunshot to the head. This may result in a markedly increased morbidity and substantial loss of function, as well as debilitating cosmesis rather than in a complete termination of life, as is the goal. This is the sad case of patient X, who is now forced to spend the rest of his life without vision, further compounding whatever underlying psychosocial stimuli initially prompted the act of attempting suicide.

## Conclusions

Failed suicide attempts may cause even more morbidity to those individuals already depressed enough to not only consider ending their life, but who attempt it with such violent means. These individuals will require significant medical and psychiatric care presumably for the rest of their lives.

## Consent

Written informed consent was obtained from the patient for publication of this case report and any accompanying images. A copy of the written consent is available for review by the Editor-in-Chief of this journal.

## Competing interests

The authors declare that they have no competing interests.

## Authors' contributions

BD and BM co-wrote and edited the manuscript. Both authors read and approved the final manuscript

## Authors' Information

Dr. Desai is the Associate Program Director for the Department of Emergency Medicine at the University of Florida.

Dr. Mahon is a second-year emergency medicine resident at the University of Florida.

